# Systematic analysis and prediction of the burden of Alzheimer’s disease and other dementias caused by hyperglycemia

**DOI:** 10.3389/fpubh.2024.1516267

**Published:** 2025-02-05

**Authors:** Dongying He, Manting Liu, Yujin Tang, Xiaona Tian, Lisi Zhou, Yizhen Chen, Xiaoxia Liu

**Affiliations:** ^1^The Eighth Clinical Medical College of Guangzhou University of Chinese Medicine, Foshan, China; ^2^Foshan Hospital of Traditional Chinese Medicine, Foshan, China; ^3^Graduate School, Guangzhou University of Chinese Medicine, Guangzhou, China; ^4^Department of Endocrinology, Zhongshan People’s Hospital (ZSPH), Zhongshan, China; ^5^Clinical Medical College, Jiangmen Wuyi Traditional Chinese Medicine Hospital, Jiangmen, China

**Keywords:** disease burden, dementia, frontier analysis, predictive analysis, Alzheimer’s disease

## Abstract

**Background:**

Alzheimer’s disease and other dementias (ADOD) pose a significant and escalating global public health challenge, particularly among the aging populations. Emerging evidence has identified high fasting plasma glucose (HFPG) as a major modifiable risk factor for ADOD, linking impaired glucose metabolism to cognitive decline and neurodegeneration. Despite this association, the comprehensive impact of HFPG on the global burden of ADOD has not been fully elucidated. Understanding the extent to which HFPG contributes to ADOD is crucial for developing targeted interventions and optimizing healthcare resource allocation to address this growing concern.

**Methods:**

Using Global Burden of Disease data (GBD 2021), we analyzed the global HFPG-related ADOD burden from 1990 to 2021 using an age-period-cohort framework and predicted trends for 2050. A Shiny platform was developed to visualize the disease burden and trends across 204 countries and regions.

**Results:**

In 2021, approximately 15% of ADOD deaths and disability-adjusted life years (DALYs) were attributed to HFPG, a 271.05% increase from 1990. The incidence rate rose from 47.07 to 66.42, indicating poor control. The mortality rate from HFPG-ADOD increased by 305.81%. The primary burden was among the 80–84 age group. Trends in ASDR and ASMR showed an increase across most SDI regions, except Low SDI. Decomposition analysis and the APC model indicated poor control in high SDI regions due to aging populations over the past 5 years. By 2050, the global prevalence is projected to reach 1,003,018.047 (26124.40377, 12114480.49), with 345,342.5738 (1431.57781, 6022119.213) males and 657,675.4731 (24692.82596, 6092361.28) females. The Shiny platform predicts a yearly increase in ASDR and ASMR for HFPG-ADOD in China, which is consistent with GBD platform trends. The platform is accessible at http://116.196.73.86:3838/GBD/HFPG-ADOD/.

**Conclusion:**

The global burden of HFPG-related ADOD has been increasing, even in high SDI regions, over the past 5 years and is expected to continue rising until 2050. Implementing appropriate health policies to mitigate this trend could significantly reduce the substantial burden caused by HFPG-induced ADOD. Promoting the use of the Shiny prediction platform will contribute significantly to global healthy aging.

## 1 Introduction

Dementia encompasses a range of progressive neurodegenerative conditions characterized by cognitive impairment, severely affecting patients’ daily activities, work capabilities, and social interactions (1). As the global population ages, dementia prevalence is expected to increase substantially; in 2018, approximately 50 million individuals worldwide were affected, and this figure is projected to rise to 152 million by 2050 (2). Alzheimer’s disease and other dementias (ADOD) have now emerged as the fifth leading cause of death globally, incurring an estimated economic loss of about US $1 trillion in 2018 (3).

Recent research has increasingly implicated diabetes as a significant comorbidity that heightens dementia risk. Large-scale epidemiological studies have reported 2- to 3-fold higher dementia incidence in individuals with diabetes compared to those without (4–6). Among the relevant metabolic factors, elevated fasting plasma glucose (HFPG) has attracted particular attention, as it can enhance oxidative phosphorylation and increase levels of the excitatory neurotransmitter glutamate, resulting in neuronal damage and cognitive dysfunction. Sustained hyperglycemia can induce glucotoxic effects, including structural and functional brain cell damage, microvascular complications leading to cerebral hemorrhage, and the accumulation of β-amyloid—hallmarks of Alzheimer’s disease (AD) pathology (7). Although cross-sectional studies generally concur that hyperglycemia is associated with cognitive decline, a clear understanding of the temporal changes, regional variations, and underlying drivers of HFPG-related Alzheimer’s disease and other dementias (HFPG-ADOD) remains lacking.

To address these critical gaps, this study leverages data from the Global Burden of Disease Study 2021 (GBD 2021) and employs an integrated age-period-cohort (APC) framework and decomposition analysis. Our objectives are to: (i) delineate the global and regional disease burden of HFPG-ADOD from 1990 to 2021, (ii) dissect the relative contributions of age, period, and cohort effects to observed trends, (iii) identify key socioeconomic, demographic, and clinical factors driving these changes, and (iv) project the future trajectory of HFPG-ADOD to 2050. By providing a more nuanced, longitudinal, and geographically granular understanding of HFPG-ADOD epidemiology, this work aims to inform evidence-based policies, guide healthcare resource allocation, and facilitate targeted interventions. Furthermore, we have developed an interactive Shiny platform to visualize current burdens and forecast future trends in 204 countries and regions, thereby empowering healthcare stakeholders, policymakers, and researchers to devise more effective prevention and management strategies.

## 2 Materials and methods

### 2.1 Data sources

This study analyzed data from the 2021 Global Burden of Disease (GBD) study, accessible via the Global Health Data Exchange (GHDx) at https://ghdx.healthdata.org. GBD 2021 provides the latest dataset on the global burden of ADOD across 204 countries and regions from 1990 to 2021. This cycle integrates new data sources and improved methods for more accurate estimates (8). Since 1990, GBD has consistently applied standard methodologies to estimate mortality and health losses for various diseases, using the Cause of Death Ensemble Model (CODEm) to estimate mortality data. This framework combines statistical models to validate cause-specific mortality (9), with results re-estimated every 2–3 years (10, 11). Mortality and disability-adjusted life years (DALYs) are derived from the average of 1,000 draws, with 95% uncertainty intervals (UI) calculated to estimate disease burden. UIs account for both parameter estimation variance and uncertainties in data collection and model selection.

### 2.2 Risk factors and definition of HFPG

In GBD 2021, the theoretical minimum risk exposure level for HFPG is 4.8–5.4 mmol/L (12). This is calculated as the person-year weighted average FPG level associated with the lowest mortality risk in meta-analyses of prospective cohort studies (13). According to the GBD framework, HFPG disease burden is only observed in individuals over 25.

### 2.3 Data analysis

For data analysis, the 2021 GBD study obtained the disease burden for 204 countries and regions, dividing the world into 21 regions based on epidemiological similarity and geographic proximity. Additionally, the Socio-demographic index (SDI), a composite indicator of social and economic conditions affecting health outcomes, is provided. Countries and regions are classified into SDI quintiles (high, high-middle, middle, low-middle, and low development levels). Age-standardized rates (ASR), mortality numbers and percentages, and DALYs were extracted from GBD 2021. EAPC of ASMR was calculated using a generalized linear model with Gaussian distribution to quantify the temporal trend of HFPG-induced ADOD. A higher EAPC indicates a heavier disease burden. If both the EAPC and its 95% confidence interval (CI) lower limit are greater than 0, the disease burden is increasing; otherwise, it indicates a declining trend. Decomposition analysis at the regional level assessed the contribution of population growth, aging, and epidemiological changes to ASDR in different SDI regions. We then used Spearman correlation analysis to examine the relationship between ASDR and SDI. Frontier analysis identified leading countries or regions (at the frontier and driving progress) with the lowest HFPG-induced ADOD burden at their SDI level. The distance from the frontier, termed “efficiency gap,” represents the gap between observed burden and potential achievable burden under its SDI; this gap may be reduced or eliminated based on the country’s or region’s socio-demographic resources. Development of Prediction Model The BAPC model assumes similar effects of age, period, and cohort in adjacent time intervals. In the BAPC model, all unknown parameters are considered random with appropriate prior distributions. The excellent predictive performance of the BAPC model has been previously validated (14, 15). The BAPC method allows us to review historical data and provides forward-looking insights for predicting future trends, aiding in public health strategy formulation. This study uses BAPC to predict HFPG-induced ADOD ASMR through 2050. HFPG-ADOD Platform Development To comprehensively assess the disease burden and BAPC predictions for 204 countries and territories, this study developed a free platform using the Shiny package (16).

### 2.4 Statistical analysis

Data analysis was conducted using R software version 4.3.2. R packages used in this study include “BAPC” (17), “tidyverse” (18), “factoextra” (19), and “ggplot2” (20). Statistical significance was considered at *p* < 0.05*.

## 3 Results

### 3.1 Global estimates of DALYs and Deaths from HFPG-induced ADOD

In 2021, approximately 15% of all ADOD DALYs were due to HFPG. The number of DALYs increased by 271.05%, from 1.44 million in 1990 to 5.35 million in 2021, doubling over the period. The incidence rate per 100,000 rose from 47.07 (2.72, 126.46) to 66.42 (3.83, 178.85), indicating ineffective control over the past three decades. The estimated annual percentage change (EAPC) was 1.175 (1.087, 1.264), reflecting a continued global increase in HFPG-ADOD DALYs, consistent with previous findings (Table 1). Similarly, nearly 15% of all ADOD-related deaths globally were attributed to HFPG, up from 10% in 1990. Deaths due to HFPG-ADOD increased by 305.81% over the past 30 years, with an EAPC of 1.198 (1.103, 1.293), indicating a significant global rise in mortality (Table 2).

**Table 1:** Burden of DALYs in HFPG-IHD population worldwide.

Location	1990			2021			EAPC
	Number	Rate	Percent	Number	Rate	Percent	
Global	1441539.86 (82703.35, 3878462.35)	47.07 (2.72, 126.46)	0.10 (0.01, 0.21)	5348853.67 (308059.6, 14351156.38)	66.42 (3.83, 178.85)	0.15 (0.01, 0.29)	1.175 (1.087, 1.264)
High SDI	565749.59 (33895.79, 1492490.69)	51.18 (3.07, 135.29)	0.11 (0.01, 0.21)	1910976.48 (112983.51, 5105646.68)	75.11 (4.45, 198.51)	0.16 (0.01, 0.32)	1.328 (1.218, 1.437)
High-middle SDI	357816.40 (20579.03, 978893.34)	46.12 (2.66, 124.91)	0.10 (0.01, 0.2)	1237594.12 (73042.77, 3272972.79)	64.33 (3.8, 170.88)	0.13 (0.01, 0.26)	1.155 (1.028, 1.283)
Middle SDI	327924.33 (17812.18, 901710.65)	48.47 (2.64, 133.72)	0.11 (0.01, 0.23)	1411109.10 (80072.24, 3799734.74)	63.35 (3.6, 172.97)	0.14 (0.01, 0.28)	0.881 (0.808, 0.955)
Low-middle SDI	142662.24 (7886.84, 378740.04)	35.92 (1.99, 95.66)	0.11 (0.01, 0.22)	622126.73 (33436.62, 1710188.64)	57.36 (3.09, 158.52)	0.16 (0.01, 0.32)	1.548 (1.519, 1.576)
Low SDI	45683.36 (2471.77, 125242.68)	34.41 (1.86, 93.92)	0.10 (0.01, 0.2)	162087.39 (8236.83, 452611.28)	49.47 (2.52, 139.57)	0.13 (0.01, 0.27)	1.197 (1.16, 1.234)
High-income Asia Pacific	106879.71 (6747.02, 271520.41)	63.12 (3.95, 161.32)	0.13 (0.01, 0.25)	451643.96 (28329.61, 1154337.64)	68.17 (4.29, 174.66)	0.15 (0.01, 0.28)	0.204 (0.117, 0.29)
High-income North America	213368.52 (13108.6, 557945.88)	57.20 (3.52, 148.96)	0.11 (0.01, 0.21)	731687.31 (43232.41, 1958233.83)	99.65 (5.91, 264.59)	0.20 (0.02, 0.39)	2.033 (1.836, 2.23)
Western Europe	282168.10 (16228.09, 751568.38)	46.35 (2.67, 124.41)	0.10 (0.01, 0.2)	779499.51 (43784.14, 2116188.93)	63.81 (3.6, 173.67)	0.14 (0.01, 0.29)	1.042 (0.976, 1.108)
Australasia	9184.02 (592.61, 24346.08)	41.29 (2.61, 109.78)	0.09 (0.01, 0.18)	33122.38 (2069.36, 86658.73)	53.45 (3.37, 140.67)	0.13 (0.01, 0.24)	0.903 (0.854, 0.951)
Andean Latin America	3923.99 (219.81, 10501.01)	23.22 (1.3, 61.77)	0.08 (0.01, 0.17)	21937.15 (1253.19, 59873.42)	39.74 (2.27, 108.36)	0.15 (0.01, 0.3)	1.85 (1.739, 1.96)
Tropical Latin America	41886.21 (2311.73, 117131.4)	61.55 (3.44, 169.07)	0.12 (0.01, 0.25)	185361.59 (10589.64, 498434.25)	76.27 (4.36, 204.86)	0.15 (0.01, 0.3)	0.851 (0.807, 0.894)
Central Latin America	32488.15 (1959.51, 85549.99)	49.62 (2.94, 130.32)	0.14 (0.01, 0.29)	137130.03 (8083.34, 365354.38)	59.09 (3.48, 158.37)	0.18 (0.01, 0.36)	0.437 (0.406, 0.468)
Southern Latin America	15031.53 (910.15, 40435.62)	37.22 (2.24, 100.3)	0.10 (0.01, 0.2)	53985.95 (3167.92, 142991.35)	58.30 (3.42, 154.79)	0.16 (0.01, 0.32)	1.438 (1.395, 1.481)
Caribbean	10331.53 (590.62, 28013.89)	45.81 (2.58, 124.65)	0.14 (0.01, 0.29)	30087.31 (1731.46, 79955.83)	54.44 (3.15, 143.84)	0.17 (0.01, 0.35)	0.495 (0.474, 0.517)
Central Europe	56072.97 (3410.54, 149742.07)	44.82 (2.72, 120.49)	0.11 (0.01, 0.22)	160789.54 (9662.7, 419378.74)	65.91 (3.96, 171.31)	0.17 (0.01, 0.34)	1.286 (1.232, 1.341)
Eastern Europe	63684.95 (3629.19, 177122.72)	27.35 (1.55, 75.53)	0.07 (0.01, 0.14)	156198.18 (8873.37, 425, 390)	42.84 (2.44, 117.18)	0.11 (0.01, 0.22)	1.642 (1.566, 1.718)
Central Asia	10000.11 (555.68, 27027.26)	26.09 (1.43, 71.86)	0.07 (0.01, 0.14)	30980.24 (1627.53, 90205.05)	50.89 (2.64, 148.85)	0.13 (0.01, 0.28)	2.495 (2.35, 2.641)
North Africa and Middle East	66255.72 (3761.14, 180360.89)	59.36 (3.36, 161.27)	0.11 (0.01, 0.23)	300181.60 (17505.76, 793107.52)	91.57 (5.33, 245.55)	0.19 (0.02, 0.39)	1.537 (1.482, 1.593)
South Asia	115263.39 (6295.07, 304136.73)	32.12 (1.76, 87.08)	0.12 (0.01, 0.24)	569301.57 (30291.75, 1590852.26)	51.08 (2.72, 142.69)	0.16 (0.01, 0.33)	1.479 (1.453, 1.506)
Southeast Asia	74193.54 (4015.75, 202005.61)	44.42 (2.34, 122.98)	0.11 (0.01, 0.23)	309893.97 (16184.35, 870213.6)	64.21 (3.31, 182.73)	0.15 (0.01, 0.32)	1.209 (1.115, 1.302)
East Asia	294747.93 (16203.49, 827599.26)	56.22 (3.1, 154.24)	0.11 (0.01, 0.21)	1246621.38 (73201.87, 3299330.89)	66.37 (3.89, 176.69)	0.12 (0.01, 0.24)	0.592 (0.432, 0.753)
Oceania	1032.33 (60.5, 2916.89)	67.24 (3.8, 189.18)	0.16 (0.01, 0.32)	3295.76 (189.22, 8938.98)	75.67 (4.18, 205.9)	0.19 (0.02, 0.38)	0.351 (0.334, 0.368)
Western Sub-Saharan Africa	14945.06 (801.89, 41308.97)	26.33 (1.4, 73.22)	0.08 (0.01, 0.18)	50610.08 (2521.41, 144778.23)	40.06 (1.99, 116.54)	0.12 (0.01, 0.26)	1.463 (1.397, 1.53)
Eastern Sub-Saharan Africa	13916.38 (703.69, 39353.53)	34.28 (1.72, 96.43)	0.08 (0.01, 0.17)	45666.46 (2148.29, 132676.22)	43.80 (2.07, 126.35)	0.09 (0.01, 0.2)	0.827 (0.792, 0.862)
Central Sub-Saharan Africa	6809.93 (388.69, 17810.16)	61.21 (3.49, 163.56)	0.11 (0.01, 0.22)	24293.42 (1295.04, 67877.25)	80.90 (4.25, 226.58)	0.14 (0.01, 0.28)	0.905 (0.842, 0.968)
Southern Sub-Saharan Africa	9355.79 (456.02, 26858.28)	47.16 (2.29, 136.25)	0.12 (0.01, 0.26)	26566.25 (1370.52, 76342.02)	64.33 (3.31, 187.21)	0.16 (0.01, 0.33)	1.218 (1.105, 1.331)

**Table 2:** Burden of Deaths in the global HFPG-IHD population.

Location	1990			2021			EAPC
	Number	Rate	Percent	Number	Rate	Percent	
Global	71470.52 (2849.13, 221701.22)	2.64 (0.11, 8.38)	0.10 (0.01, 0.21)	290032.19 (11759.52, 916714.18)	3.73 (0.15, 11.84)	0.15 (0.01, 0.29)	1.198 (1.103, 1.293)
High SDI	30545.19 (1283.05, 95702.96)	2.88 (0.12, 9.17)	0.11 (0.01, 0.21)	116114.61 (4820.68, 364124.07)	4.27 (0.18, 13.27)	0.16 (0.01, 0.32)	1.362 (1.248, 1.476)
High-middle SDI	17555.54 (696.59, 55919.93)	2.61 (0.1, 8.38)	0.10 (0.01, 0.2)	65959.34 (2680.76, 205256.98)	3.54 (0.14, 11.11)	0.13 (0.01, 0.26)	1.087 (0.944, 1.23)
Middle SDI	14869.26 (563.44, 48060.03)	2.66 (0.1, 8.58)	0.11 (0.01, 0.23)	69334.61 (2706.71, 219022.4)	3.42 (0.13, 10.92)	0.14 (0.01, 0.28)	0.868 (0.795, 0.942)
Low-middle SDI	6394.09 (234.99, 20480.52)	1.92 (0.07, 6.09)	0.11 (0.01, 0.22)	30601.01 (1133.95, 98703.47)	3.19 (0.12, 10.43)	0.16 (0.01, 0.32)	1.721 (1.683, 1.758)
Low SDI	2025.29 (73.88, 6573.13)	1.91 (0.07, 6.19)	0.10 (0.01, 0.2)	7756.94 (280.13, 25000.78)	2.83 (0.1, 9.31)	0.13 (0.01, 0.26)	1.335 (1.275, 1.396)
High-income Asia Pacific	5806.70 (260.34, 17464.79)	3.78 (0.17, 11.46)	0.13 (0.01, 0.26)	29847.51 (1408.95, 88268.89)	4.00 (0.19, 11.88)	0.15 (0.01, 0.28)	0.126 (0.066, 0.186)
High-income North America	11460.44 (500.34, 35027.62)	3.09 (0.14, 9.47)	0.11 (0.01, 0.2)	43128.15 (1762.04, 137444.53)	5.66 (0.23, 18.07)	0.20 (0.02, 0.4)	2.228 (2.018, 2.439)
Western Europe	15706.32 (624.24, 49391.8)	2.69 (0.11, 8.61)	0.10 (0.01, 0.2)	48689.92 (1947.18, 153966.43)	3.71 (0.15, 11.65)	0.14 (0.01, 0.29)	1.065 (0.996, 1.134)
Australasia	470.23 (19.97, 1493.45)	2.26 (0.1, 7.24)	0.09 (0.01, 0.18)	1953.64 (84.16, 5953.43)	3.00 (0.13, 9.17)	0.13 (0.01, 0.24)	1.002 (0.95, 1.055)
Andean Latin America	192.78 (7.33, 635.14)	1.21 (0.05, 3.99)	0.08 (0.01, 0.17)	1124.46 (45.45, 3659.78)	2.07 (0.08, 6.73)	0.15 (0.01, 0.3)	1.851 (1.734, 1.968)
Tropical Latin America	1954.77 (76.98, 6265.15)	3.31 (0.13, 10.48)	0.12 (0.01, 0.24)	9627.75 (403.35, 29678.83)	4.04 (0.17, 12.44)	0.15 (0.01, 0.29)	0.833 (0.778, 0.888)
Central Latin America	1412.61 (54.35, 4587.86)	2.40 (0.09, 7.84)	0.14 (0.01, 0.28)	6645.31 (261.07, 21540.04)	2.91 (0.11, 9.44)	0.17 (0.01, 0.36)	0.465 (0.424, 0.506)
Southern Latin America	734.16 (28.32, 2414.71)	1.99 (0.08, 6.51)	0.10 (0.01, 0.19)	2949.08 (116.33, 9427.4)	3.15 (0.12, 10.08)	0.16 (0.01, 0.31)	1.49 (1.45, 1.531)
Caribbean	477.44 (17.76, 1576.04)	2.34 (0.09, 7.89)	0.14 (0.01, 0.29)	1575.87 (61.67, 5158.35)	2.77 (0.11, 9.07)	0.17 (0.01, 0.35)	0.523 (0.501, 0.545)
Central Europe	2626.34 (104.41, 8449.87)	2.39 (0.09, 7.83)	0.11 (0.01, 0.22)	8628.00 (348.03, 27517.63)	3.52 (0.14, 11.21)	0.17 (0.01, 0.34)	1.291 (1.242, 1.34)
Eastern Europe	2968.93 (112.33, 9581.87)	1.46 (0.05, 4.81)	0.07 (0.01, 0.14)	8180.52 (307.65, 26022.7)	2.27 (0.08, 7.25)	0.11 (0.01, 0.22)	1.625 (1.548, 1.702)
Central Asia	490.87 (19.11, 1627.26)	1.39 (0.05, 4.67)	0.07 (0.01, 0.14)	1476.55 (50.51, 4918)	2.67 (0.09, 9.01)	0.13 (0.01, 0.28)	2.436 (2.28, 2.592)
North Africa and Middle East	3068.49 (119.15, 9853.56)	3.20 (0.13, 10.32)	0.11 (0.01, 0.23)	14262.12 (573.58, 45460.06)	4.89 (0.19, 15.67)	0.19 (0.02, 0.38)	1.509 (1.443, 1.576)
South Asia	5029.46 (176.31, 16404.71)	1.71 (0.06, 5.56)	0.12 (0.01, 0.25)	27989.16 (1029.02, 90775.73)	2.87 (0.1, 9.42)	0.17 (0.01, 0.34)	1.737 (1.702, 1.771)
Southeast Asia	3447.53 (126.2, 11276.46)	2.40 (0.09, 7.87)	0.12 (0.01, 0.25)	15379.75 (568.34, 51423.53)	3.58 (0.13, 11.99)	0.16 (0.01, 0.34)	1.296 (1.182, 1.411)
East Asia	13420.49 (534.13, 44749.91)	3.31 (0.13, 10.45)	0.11 (0.01, 0.21)	61211.94 (2425.89, 189557.04)	3.62 (0.14, 11.46)	0.12 (0.01, 0.24)	0.395 (0.236, 0.555)
Oceania	41.07 (1.61, 135.31)	3.60 (0.14, 12.1)	0.16 (0.01, 0.32)	140.24 (5.15, 463.27)	4.01 (0.15, 13.44)	0.19 (0.02, 0.39)	0.317 (0.301, 0.334)
Western Sub-Saharan Africa	742.27 (28.09, 2437.49)	1.55 (0.06, 5.12)	0.09 (0.01, 0.18)	2545.22 (87.75, 8558.91)	2.36 (0.08, 8.03)	0.12 (0.01, 0.25)	1.475 (1.401, 1.549)
Eastern Sub-Saharan Africa	659.16 (24.12, 2193.96)	2.02 (0.07, 6.75)	0.08 (0.01, 0.18)	2282.06 (77.66, 7466.43)	2.57 (0.09, 8.6)	0.09 (0.01, 0.2)	0.835 (0.806, 0.863)
Central Sub-Saharan Africa	285.77 (12.05, 888.17)	3.53 (0.15, 11.07)	0.12 (0.01, 0.23)	1124.61 (43.08, 3565.3)	4.70 (0.18, 15.26)	0.13 (0.01, 0.27)	0.93 (0.846, 1.014)
Southern Sub-Saharan Africa	474.70 (16.22, 1570.02)	2.64 (0.09, 8.79)	0.12 (0.01, 0.27)	1270.30 (45.27, 4343.93)	3.56 (0.12, 12.35)	0.16 (0.01, 0.33)	1.136 (1.018, 1.254)

### 3.2 Regional burden of HFPG-ADOD

**Figure 1:**
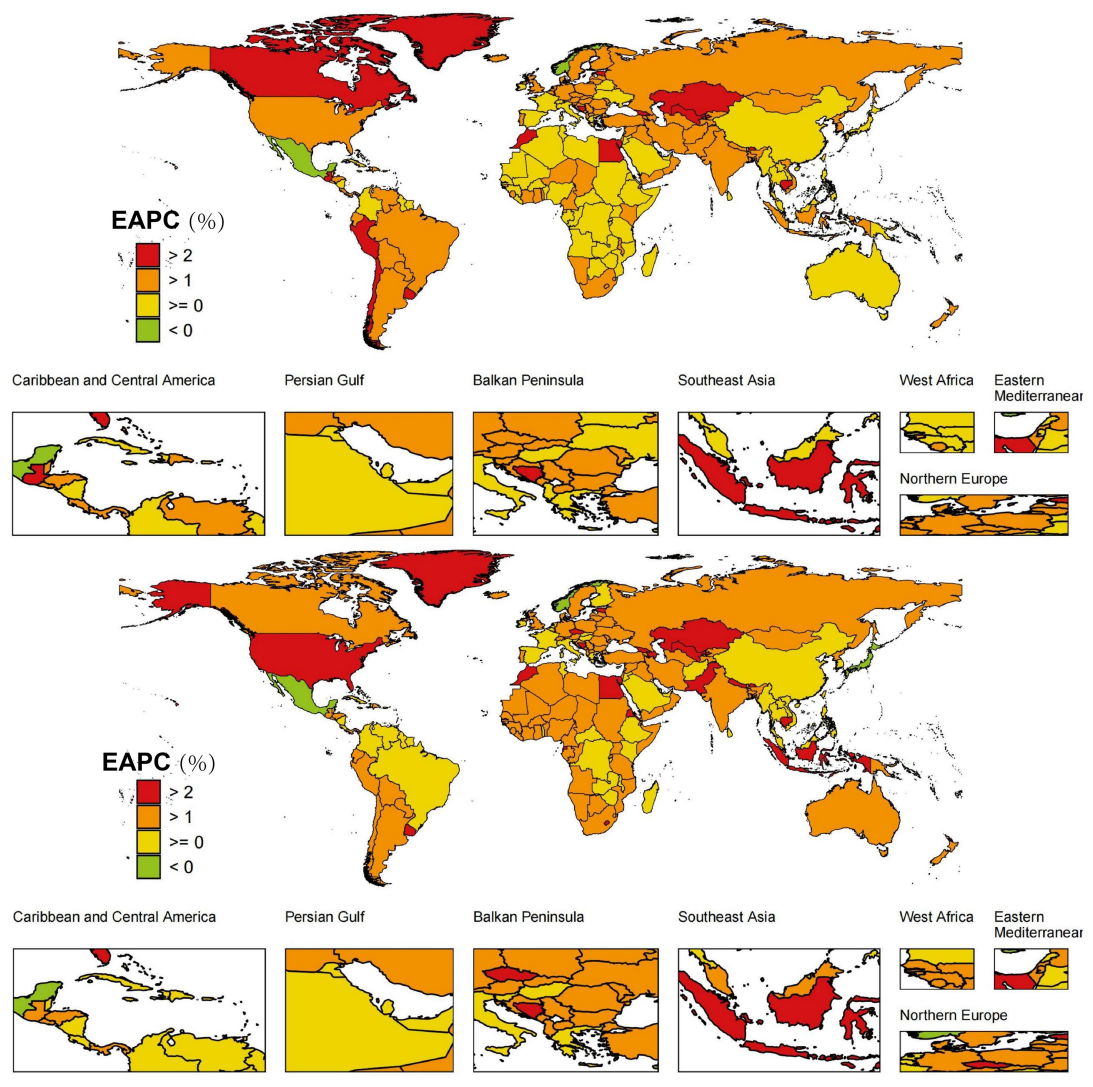
**(A)** Global distribution of ASMR trends (EAPC) in male HFPG-IHD population; **(B)** Global distribution of ASMR trends (EAPC) in female HFPG-IHD population.

The DALYs and mortality burden of HFPG-ADOD have generally increased globally, with regional differences. By SDI classification, both High SDI [DALYs EAPC: 1.175 (1.087, 1.264), ASMR EAPC: 1.198 (1.103, 1.293)] and Low SDI [DALYs EAPC: 1.197 (1.16, 1.234), ASMR EAPC: 1.335 (1.275, 1.396)] regions showed similar trends (Tables 1, 2). Further gender-based analysis revealed consistent ASMR EAPC trends across genders, with regional variations such as in North Africa (Figure 1), warranting further stratified analysis.

### 3.3 Disease burden by gender

**Figure 2:**
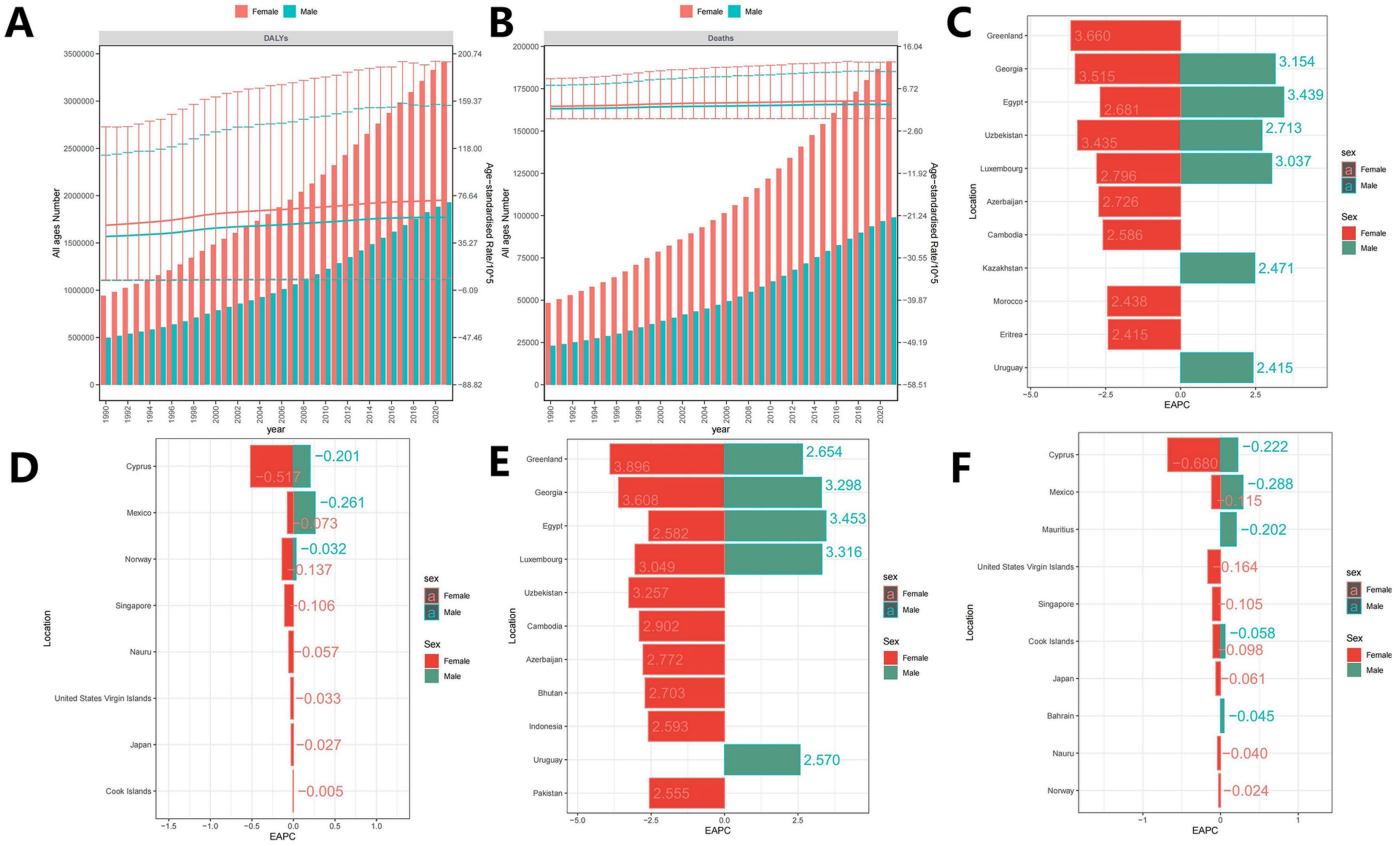
**(A,B)** Annual trend of global ASDR and ASMR (Note: **A** is DALYs burden, bar chart represents DALYs number, line chart represents ASDR; **B** is the burden of death, the bars represent the number of deaths, and the line graphs represent the ASMR); **(C)** Top 10 regions with the fastest increasing trends in ASMR (EAPC); **(D)** Top 10 regions with the fastest decreasing trend in ASMR (EAPC); **(E)** Top 10 regions with the highest ASMR; **(F)** Regional populations in the top 10 lowest ASMR.

Over the past 30 years, the DALYs and mortality burden for both male and female patients have increased. Notably, ASDR and ASMR were similar across genders (Figure 2A). Given the global variability in HFPG-ADOD ASDR and ASMR burdens, we identified regions and genders with the highest growth and best mitigation. For ASDR growth, Greenland’s female population observed the highest increase [EAPC: 3.66 (3.14, 4.18)], followed by females in Georgia [EAPC: 3.52 (3.21, 3.83)], and males in Egypt [EAPC: 3.44 (3.16, 3.72)] and females in Uzbekistan [EAPC: 3.43 (3.19, 3.68)], with top regions having a 2021 SDI of 0.40–0.88 (Figure 2B). Some regions achieved negative ASDR growth, including females in Cyprus [EAPC: −0.52 (−0.56, −0.48)], males in Mexico [EAPC: 0.26 (−0.3, −0.23)], and males in Cyprus [EAPC: −0.2 (−0.29, −0.12); (Figure 2C], with top regions having a 2021 SDI of 0.62–0.91, higher than regions with ASDR growth. In ASMR growth rankings, Greenland’s female population observed the highest increase [EAPC: 3.90 (3.32, 4.47)], followed by females in Georgia [EAPC: 3.61 (3.28, 3.94)] and males in Egypt [EAPC: 3.45 (3.16, 3.74)], consistent with ASDR rankings. Top regions had a 2021 SDI of 0.47–0.88 (Figure 2D). Some regions achieved negative ASDR growth, including females in Cyprus [EAPC: −0.68 (−0.75, −0.61)], males in Mexico [EAPC: 0.29 (−0.33, −0.25)], and males in Cyprus [EAPC: −0.22 (−0.33, −0.12); Figure 2E], with top regions having a 2021 SDI of 0.63–0.91, higher than regions with ASDR growth, consistent with ASDR trends.

### 3.4 Disease burden by age group

**Figure 3:**
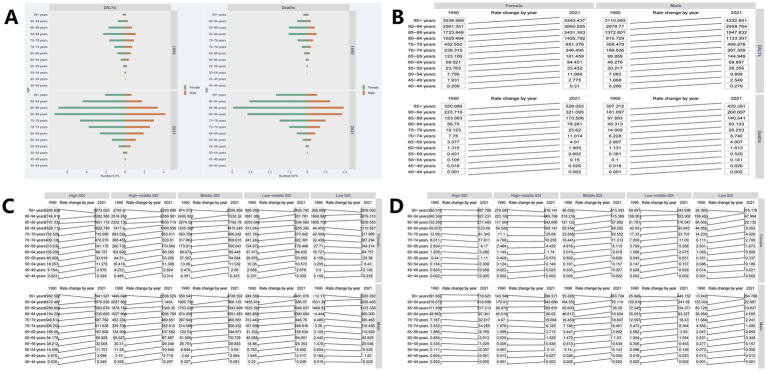
**(A)** Global burden of DALYs, Deaths by age group; **(B)** Trends in the global burden of DALYs and Deaths by age; **(C)** Trends of ASDR in different age groups during 1990 to 2021 in different SDI regions; **(D)** Trends of ASMR in different age groups from 1990 to 2021 in different SDI regions.

**Figure 4:**
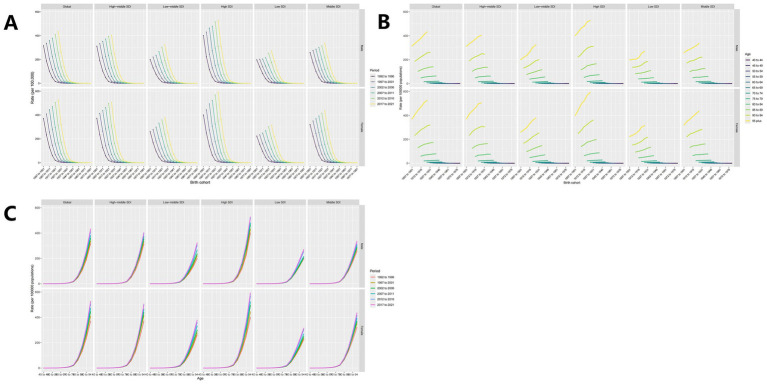
**(A)** Perid-cohort effect; **(B)** Age-Period effect; **(C)** Age-cohort effect.

In this study, the DALYs burden for HFPG-ADOD in 1990 was primarily in the 80–84 age group for both males and females. By 2021, this age group remained the main burden, with a decrease on either side (Figure 3A). The mortality burden shifted to the 85–89 age group, older than the 80–84 group in 1990, with significant increases in DALYs and deaths (Figure 3A). Age-adjusted ASDR and ASMR also showed a rising trend (Figure 3B). We further analyzed mortality burden by SDI region. Over the past 30 years, ASDR increased in High, High-middle, Middle, and Low-middle SDI regions, except Low SDI (Figure 3C). ASMR trends rose in High-middle, Middle, Low-middle, and Low SDI regions, except High SDI (Figure 3D). Age, Period, and Cohort Effects on ASDR The APC model estimated the effects of age, period, and cohort by SDI quintiles, evaluating their contribution to ASMR in different SDI regions. Age effects are shown by longitudinal age curves, describing ASMR’s natural history. Period effects indicate changing relative risks over time, tracking progress. Cohort effects show relative risk changes in birth cohorts. Overall, age, period, and cohort effects showed consistent trends globally and across SDI regions. The period-cohort model indicated a global similarity in birth cohort effects, generally declining (Figure 4A). Period effects were more pronounced, with ASDR gradually decreasing over 30 years without clear shifts (Figure 4A). The age-period cohort model (Figure 4B) confirmed that higher age correlates with higher ASDR, as shown in the age-period model (Figure 4C), highlighting age as a key factor in HFPG-induced ADOD mortality burden. Notably, in the past 5 years, ASDR showed the highest ASMR globally and across SDI regions (Figure 4C), suggesting recent medical advancements have not been effectively realized.

### 3.5 Correlation analysis of SDI and ASMR

**Figure 5:**
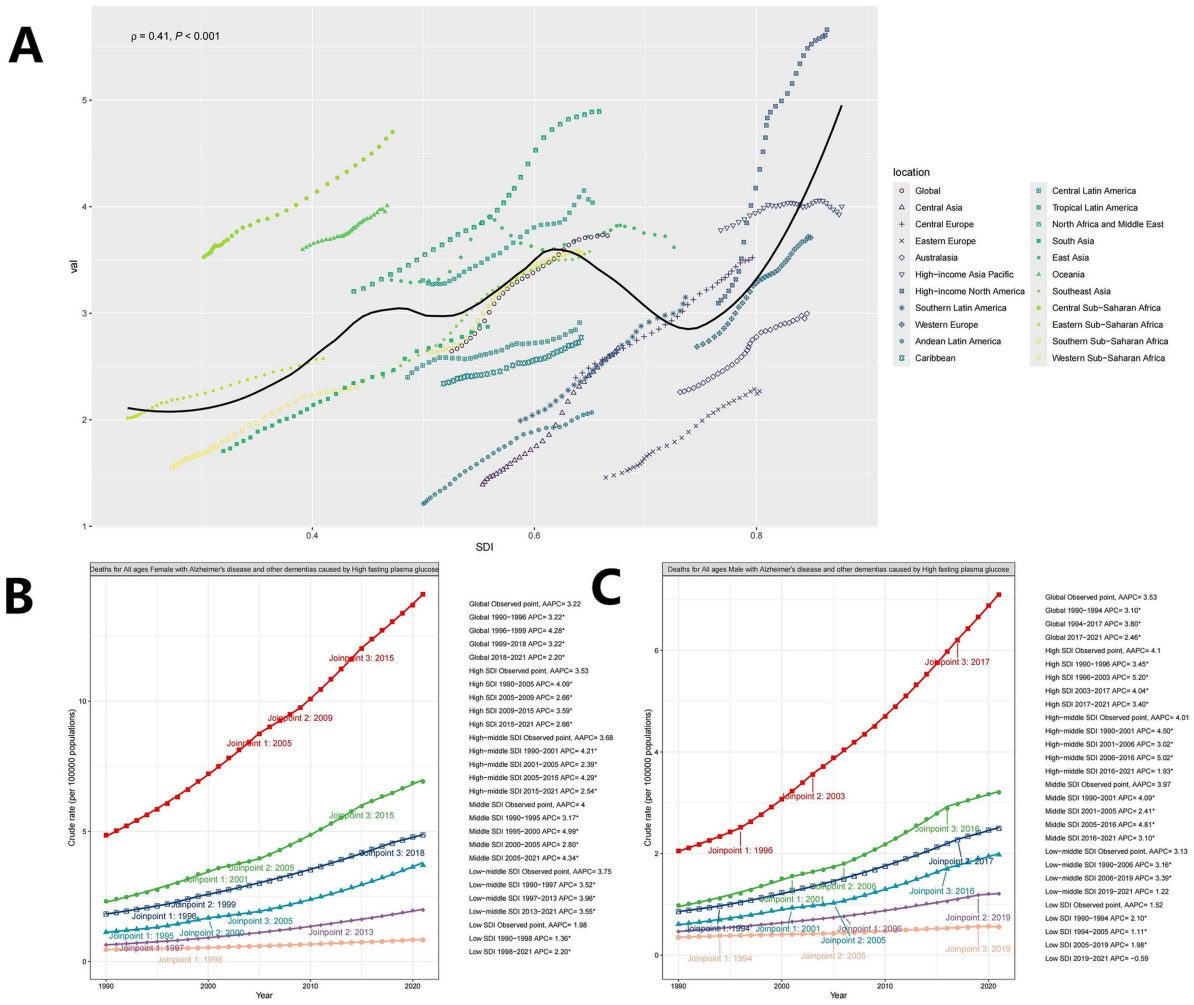
**(A)** Scatter plot of correlation between ASMR and SDI (Note: Points represent the mapping of SDI and ASMR for each location in each year from 1990 to 2021, and the curve represents the fitting line); **(B)** Deaths for All ages Female with AD and other dementias caused by HFPG; **(C)** Deaths for All ages Male with AD and other dementias caused by HFPG.

To further validate the correlation between ASMR and SDI, Spearman correlation analysis showed a positive relationship globally (cor = −0.142, *p* = 0.000 < 0.001; Figure 5A). Using a Joinpoint AAPC model, ASMR has shown a continuous upward trend over the past 30 years, consistent across both male (Figure 5B) and female (Figure 5C) populations.

### 3.6 Frontier analysis

**Figure 6:**
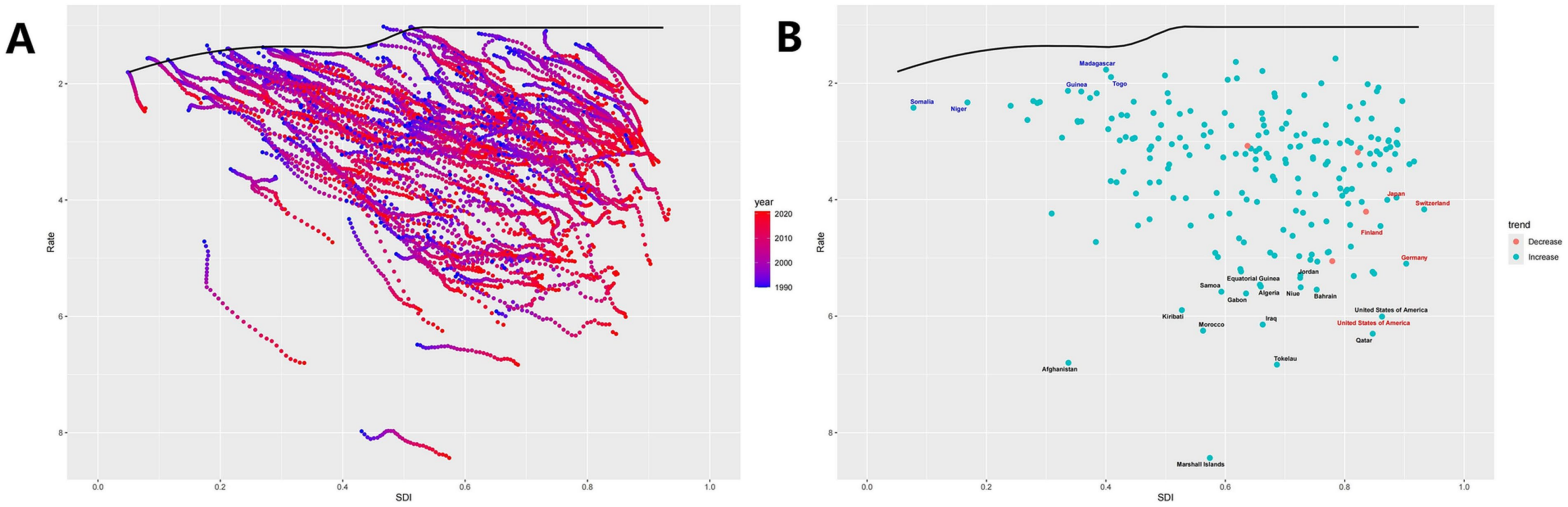
Frontier analysis. **(A)** Plot of SDI versus ASMR for each region for each year from 1990 to 2021. The dots in the figure represent the SDI versus ASMR for each region in each year during this period, and the curves represent the ideal ASMR at a specific SDI. **(B)** Plot of the change trend of ASMR in 2021 compared with 1990 in each region. The midpoint of the figure shows the trend of ASMR in 2021 for each region compared with 1990, where the blue indicates the increase, the red indicates the decrease, and the curve represents the ideal ASMR at the specified SDI.

Overall, as SDI increases, the efficiency gap tends to widen, indicating less effective ASMR control in high SDI regions (Figure 6A). The top five countries with the largest efficiency gaps compared to the frontier include Morocco, Qatar, Afghanistan, Tokelau, and the Marshall Islands (range: 5.21–7.40), showing significantly higher ASMR at similar SDI levels. Conversely, the top five countries with the lowest ASDR and smallest efficiency gaps at similar SDI levels (range: 0.42–0.62) are Madagascar, Belarus, Togo, Mongolia, and Somalia (Figure 6B).

### 3.7 Decomposition analysis

**Figure 7:**
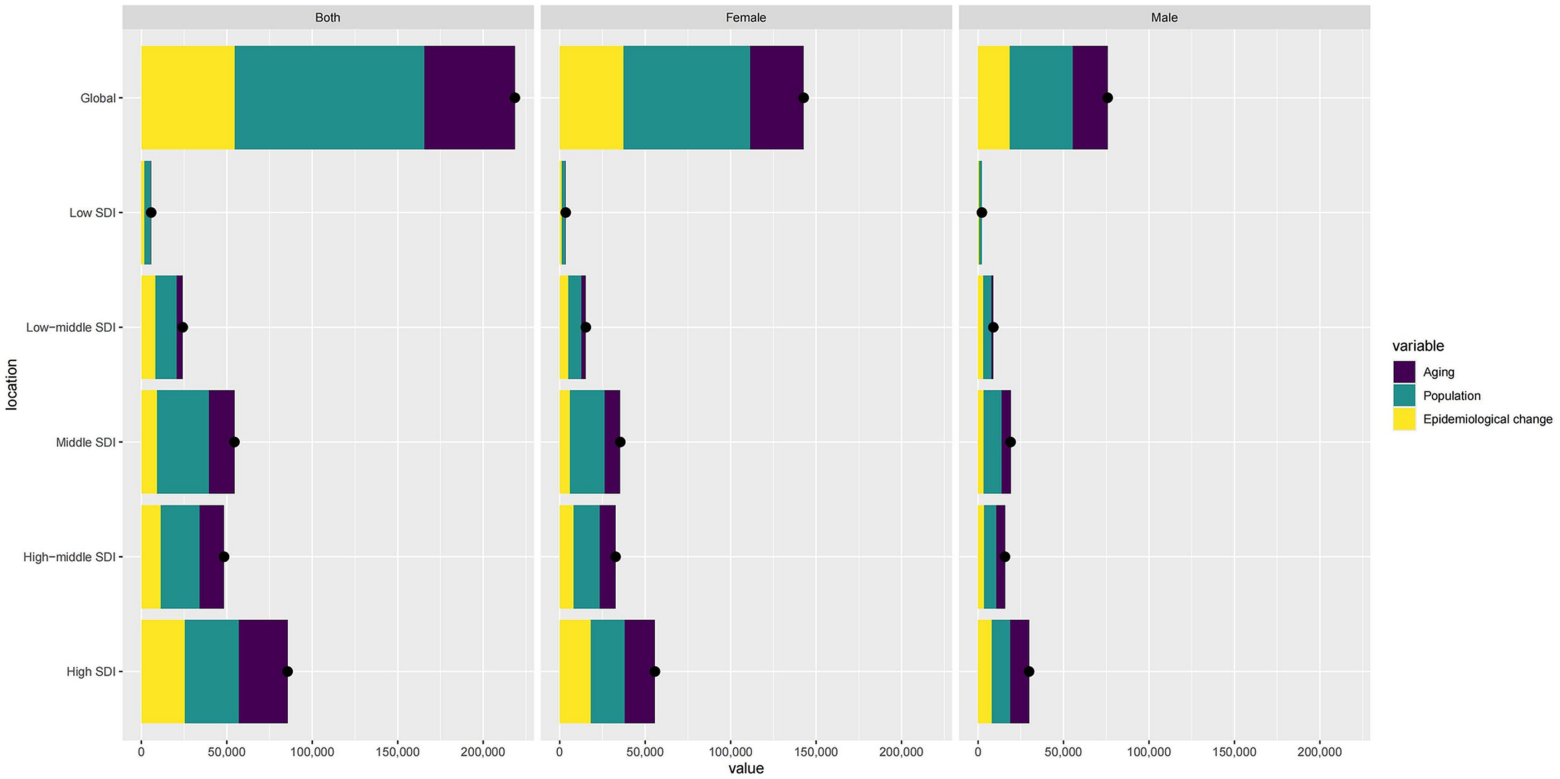
Comparison of contribution values of Aging, Population and Epidemiological change based on different genders (Note: bars represent specific contribution values, points represent relative proportions).

Globally, population growth is the main contributor to ASMR, followed by epidemiological changes and aging. These factors show regional differences: in Low and Low-middle SDI areas, population is the largest contributor; starting from Middle SDI, aging becomes more significant. In High SDI regions, aging’s impact is close to population, with epidemiological changes surpassing population growth (Figure 7).

### 3.8 BAPC predictions

**Figure 8:**
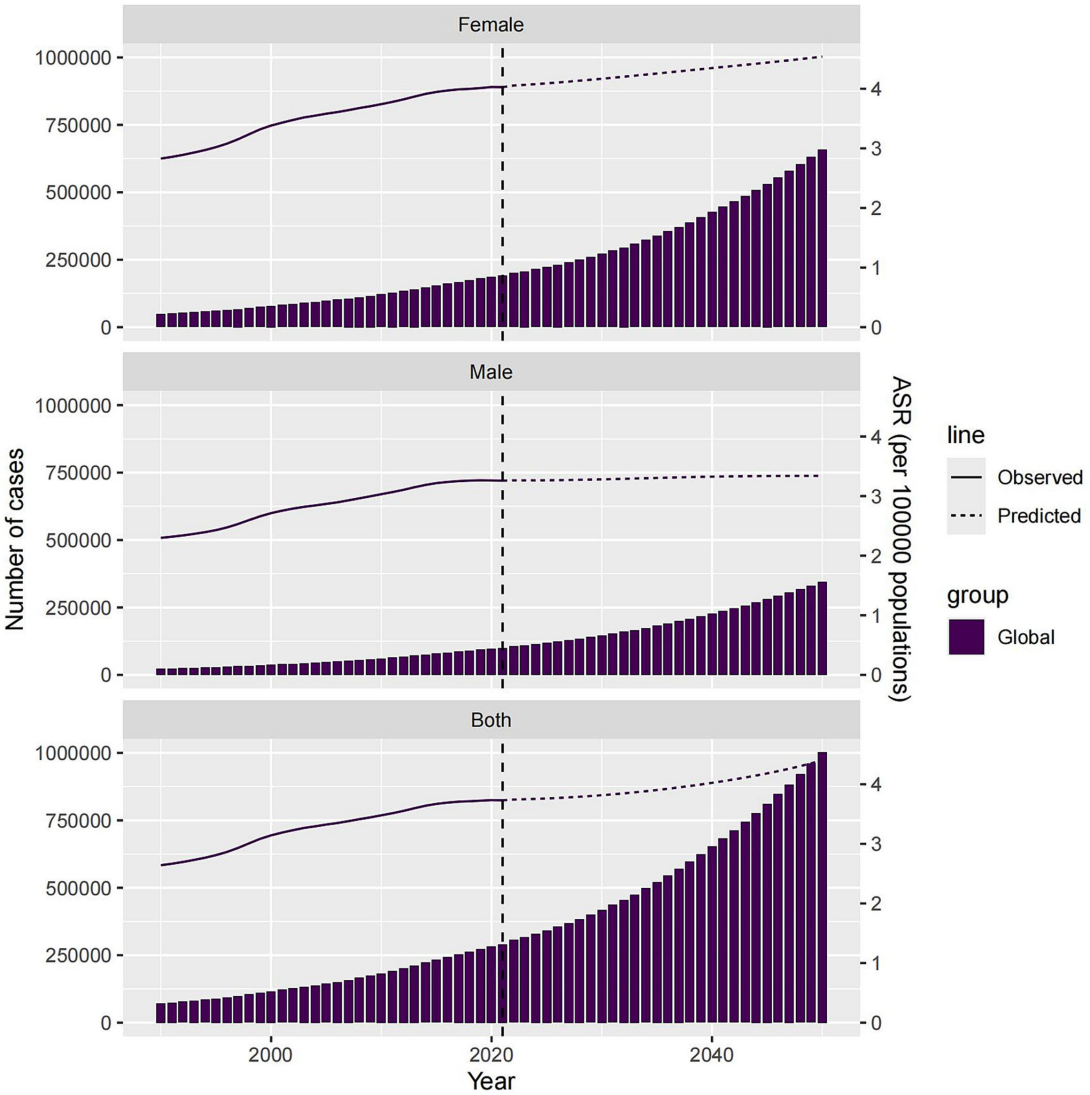
Number of Deaths and ASMR by gender predicted for 2050 globally and in China.

Finally, using BAPC, we predicted global HFPG-ADOD ASMR from 2022 to 2050 and conducted gender subgroup analysis. By 2050, the absolute number of cases is projected to rise to 1,003,018.047 (26,124.40377, 12,114,480.49), with males at 345,342.5738 (1,431.57781, 6,022,119.213) and females at 657,675.4731 (24,692.82596, 6,092,361.28). ASMR is projected to rise to 4.40 (0.09, 84.53), with a gradual increase, while male ASMR is 4.53 (0.94, 8.14), showing a moderate trend. It indicates that females will contribute most to the global ASDR increase over the next 30 years (Figure 8).

### 3.9 Shiny platform development

**Figure 9:**
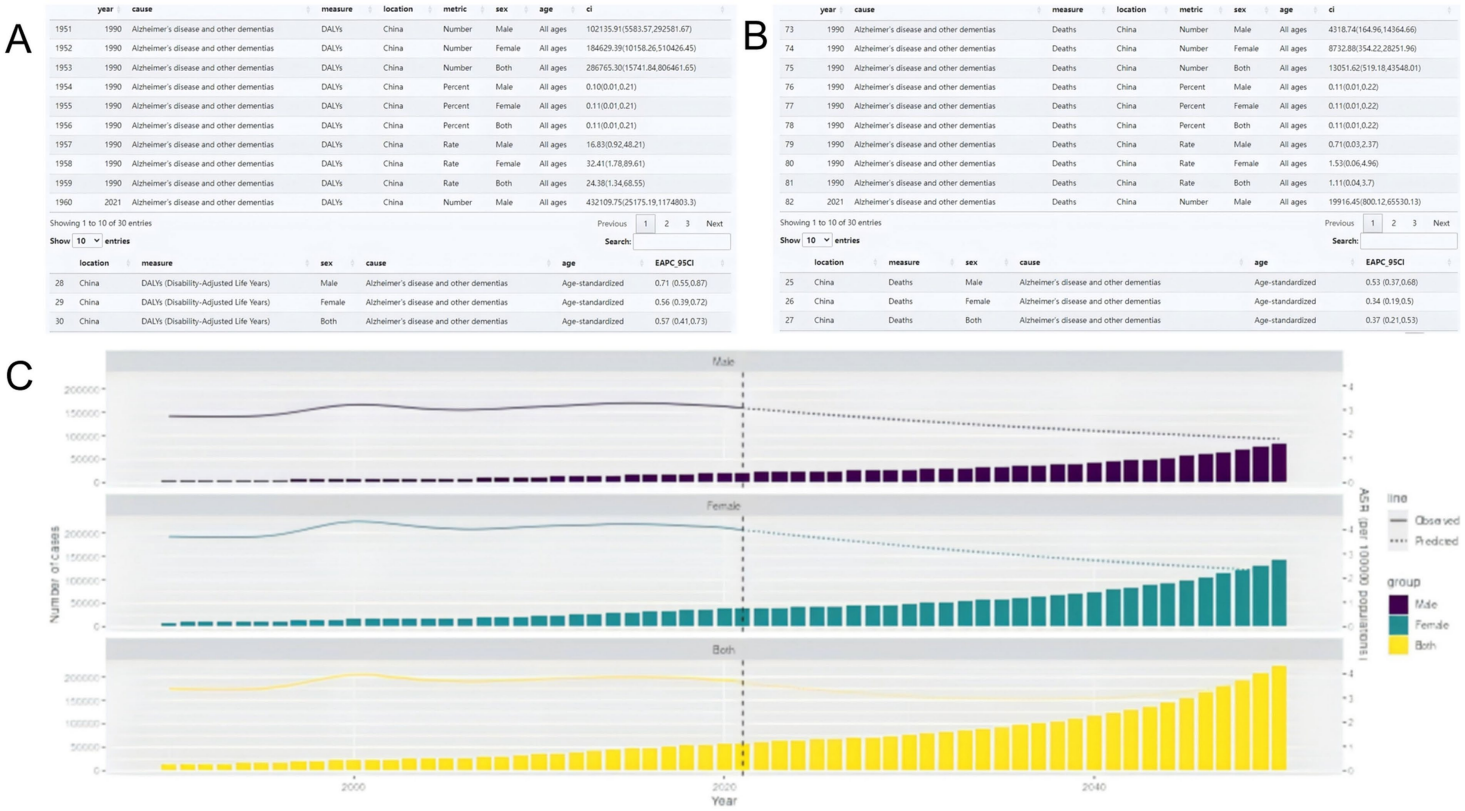
**(A)** HFPG-IHD platform construct; **(B)** Disease burden table; **(C)** Predicted trends.

Using the Shiny platform, we developed the HFPG-ADOD DATABASE to visualize disease burden and predictions for 204 countries and regions, accessible at http://116.196.73.86:3838/GBD/HFPG-ADOD/. For example, in China, EAPC analysis shows an annual increase in ASDR [EAPC: 0.57 (0.41, 0.73); (Figure 9A], and ASMR also shows an increasing trend [EAPC: 0.37 (0.21, 0.53); Figure 9B]. Based on BAPC predictions, by 2050, HFPG-ADOD deaths will rise from 13,039.81 (5,592.12, 109,990.79) to 224,964.46 (0, 2,257,566.33), but ASMR is expected to decrease [ASMR: 3.96 (0, 82.31); Figure 9C].

## 4 Discussion

Diabetes, characterized by chronic hyperglycemia, is a metabolic disorder affecting 537 million people globally in 2021, according to the International Diabetes Federation. This means over 1 in 10 adults has diabetes. The International Diabetes Federation and WHO project this number to rise to 643 million by 2030 (21). Thus, diabetes poses a significant public health challenge. There is a strong link between type 2 diabetes and increased dementia risk, with HFPG playing a crucial role. HFPG is associated with severe long-term complications, including ADOD, leading researchers to term AD as “type 3 diabetes” (22, 23). The disease burden from HFPG-ADOD is a global concern. This study aims to explore trends and drivers of HFPG-ADOD burden across regions and predict changes by 2050, providing a visual platform for different countries to support better healthcare resource allocation and policy-making. Although studies indicate a decline in AD incidence in high-income countries (24), we observe an alarming increase in global DALYs and deaths from HFPG-ADOD, with deaths rising by 305.81%. The EAPC values of 1.175 (1.087, 1.264) and 1.198 (1.103, 1.293) suggest that advancements in medical technology over the past 30 years have not effectively controlled the disease burden from HFPG-ADOD.

Despite studies showing a decline in AD incidence in high-income countries (24), we observe a continuous rise in global DALYs and deaths due to HFPG-ADOD, with deaths increasing by 305.81%. This is concerning, as EAPC values of 1.175 (1.087, 1.264) and 1.198 (1.103, 1.293) suggest that technological advancements over 30 years have not effectively reduced the disease burden. In 2012, Banerjee et al. (25) noted that half of dementia patients lived in high-income countries, 39% in middle-income, and 14% in low-income countries. By 2023, cases are expected to rise significantly in low and middle-income countries, with prevalence increasing linearly in high-income and exponentially in low-income areas. Walsh et al. (26) also predict a rise in dementia, especially in low and middle-income regions. In high-income areas, the prevalence of prediabetes and diabetes has surged, largely driven by obesity (27, 28), leading to increased HFPG ASDR. This contradiction results in similar burdens across different SDI levels, with consistent ASDR and ASMR trends and similar burdens between genders. To further explore the influence of age, cohort, and period across SDI regions, we developed an APC model. It shows that age is a key factor in HFPG-ADOD mortality. Notably, the past 5 years have seen the highest ASMR globally and across SDI regions, with a positive correlation between SDI and ASMR. This suggests that high SDI benefits do not translate into reduced HFPG-ADOD burden. High SDI regions may have higher sugar intake and lower physical activity, offsetting healthcare improvements (29), while low SDI regions may lack medical resources. Our decomposition analysis reveals that in Low and Low-middle SDI areas, population growth is the largest contributor, aligning with previous studies. From Middle SDI, aging becomes more significant, and in High SDI regions, the impact of aging approaches population growth, with epidemiological changes surpassing it. This aligns with ADOD as an age-related disease. Tailored health policies are needed: high SDI areas should encourage balanced diets and regular exercise, with early screening for diabetes and dementia; middle SDI areas should enhance public health education and support groups; low SDI areas need to build primary healthcare capacity and promote sustainable agriculture and exercise programs. Our predictions indicate that by 2050, global cases will rise to 1,003,018.047 (26,124.40377, 12,114,480.49), with males at 345,342.5738 (1,431.57781, 6,022,119.213) and females at 657,675.4731 (24,692.82596, 6,092,361.28), with females contributing more to the ASDR increase. Economic and policy differences hinder HFPG-ADOD assessment, so we developed a personalized visualization platform to evaluate burden and trends, enhancing policy implementation. In summary, from 1990 to 2021, the global HFPG-ADOD burden has increased, affecting most regions and impacting quality of life. Despite longer lifespans and technological advances, even high SDI regions struggle to improve this situation. By 2050, cases are projected to reach 1 million, posing significant health and economic challenges. Evaluating the cost-effectiveness of prevention and treatment, optimizing resources, and strengthening early screening and intervention for high-burden groups are essential.

## Data Availability

The raw data supporting the conclusions of this article will be made available by the authors, without undue reservation.
